# Supplementary material for: Interpreting biologically informed neural networks for enhanced proteomic biomarker discovery and pathway analysis
Source: Nat Commun. 2023 Sep 2;14:5359. doi: 10.1038/s41467-023-41146-4 (PMC10475049; doi:10.1038/s41467-023-41146-4)
Supplement: Supplementary file 1 — Supplementary Figures [file 41467_2023_41146_MOESM1_ESM.pdf]

Interpreting biologically informed neural networks for enhanced proteomic biomarker discovery and pathway analysis

Erik Hartman[1,*], Aaron Scott[1], Christofer Karlsson[1], Tirthankar Mohanty[1], Suvi T. Vaara[2], Adam Linder [1], Lars Malmström[1] and Johan Malmström[1,*]

[1]Division of Infection Medicine, Department of Clinical Sciences Lund, Faculty of Medicine, Lund University, Lund, Sweden

[2]Department of Perioperative and Intensive Care, University of Helsinki and Helsinki University Hospital, Helsinki, Finland

*Corresponding authors: Erik Hartman (erik.hartman@hotmail.com), Johan Malmström (johan.malmstrom@med.lu.se)

Erik Hartman and Aaron Scott contributed equally to the manuscript.

Keywords: proteomics, deep learning, biomarker identification, biological pathway analysis

Abstract

The incorporation of machine learning methods into proteomics workflows improves the identification of disease-relevant biomarkers and biological pathways. However, machine learning models, such as deep neural networks, typically suffer from lack of interpretability. Here, we present a deep learning approach to combine biological pathway analysis and biomarker identification to increase the interpretability of proteomics experiments. Our approach integrates *a priori* knowledge of the relationships between proteins and biological pathways and biological processes into sparse neural networks to create *biologically informed neural networks*. We employ these networks to differentiate between clinical subphenotypes of septic acute kidney injury and COVID-19, as well as acute respiratory distress syndrome of different aetiologies. To gain biological insight into the complex syndromes, we utilize feature attribution-methods to introspect the networks for the identification of proteins and pathways important for distinguishing between subtypes. The algorithms are implemented in a freely available open source Python-package (`https://github.com/InfectionMedicineProteomics/BINN`).

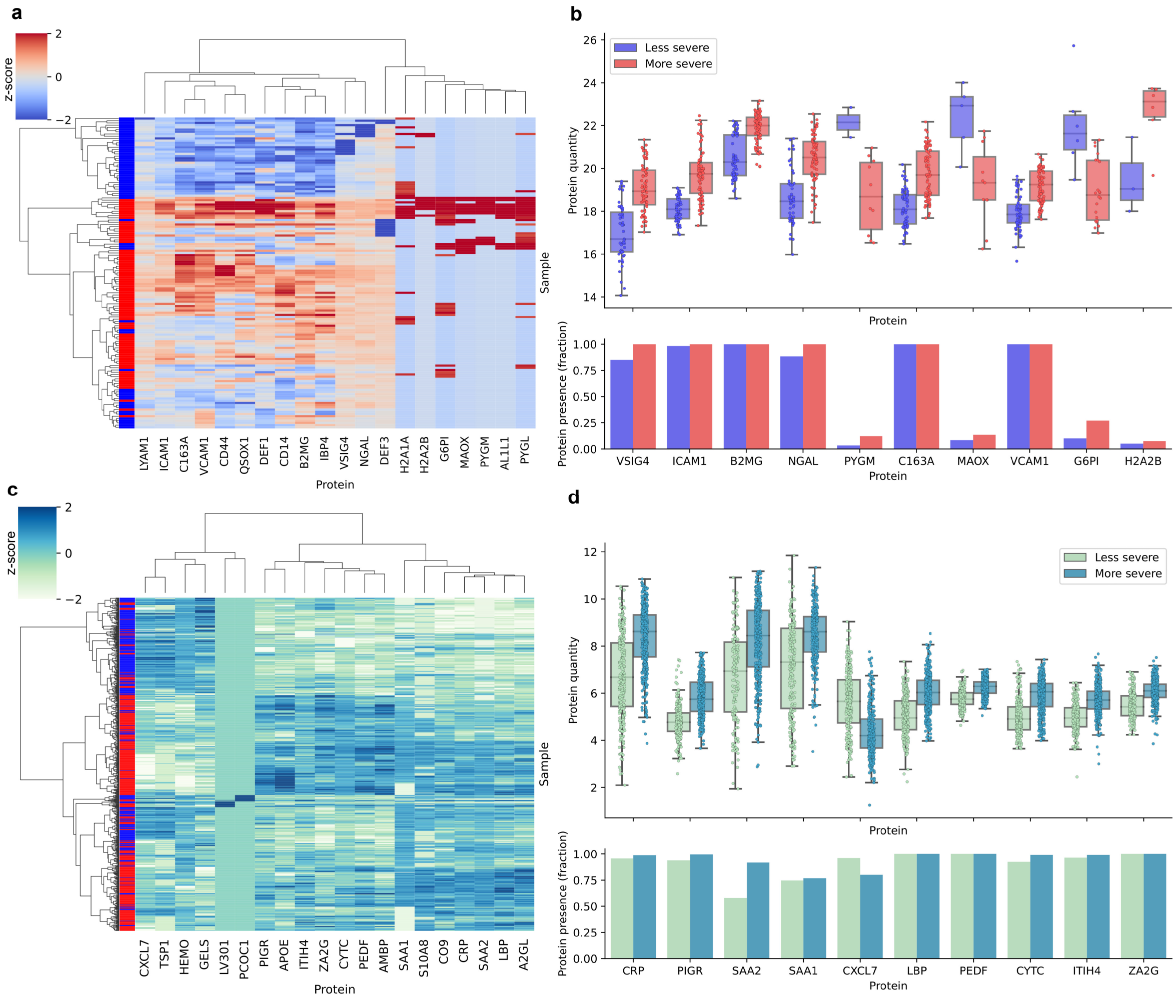

Supplementary Figure 1: The most important proteins in the septic AKI and COVID-BINNs by DE-score. The DE-score is defined in **equation 4**. The most important proteins as defined by DE-score were selected and subject to hierarchical clustering. **a)** A clustermap showcasing the clustering based on the scaled protein abundances of the top 20 proteins with the highest DE-score in the sepsis dataset. The left-most column shows the subphenotype classification (subphenotype 2: red, subphenotype 1: blue). Clustering was performed using Wards minimum variance method and Euclidean distances. The Rand-index for the clustering was 0.716. **b)** The upper panel shows the protein quantity for the 10 proteins with highest DE-score. The boxes show the quartiles of the dataset while the whiskers extend to show the rest of the distribution, except for points that are determined to be “outliers” using a method that is a function of the inter-quartile range. The center-line shows the mean of the dataset. *n=141* biologically independent samples. The lower panel shows which fraction of samples identified the given protein. **c)** Same as **a** but on the COVID-dataset. The Rand-index for the clustering was 0.645. **d)** same as **b** but for the COVID-dataset. Here *n=687* biologically independent samples

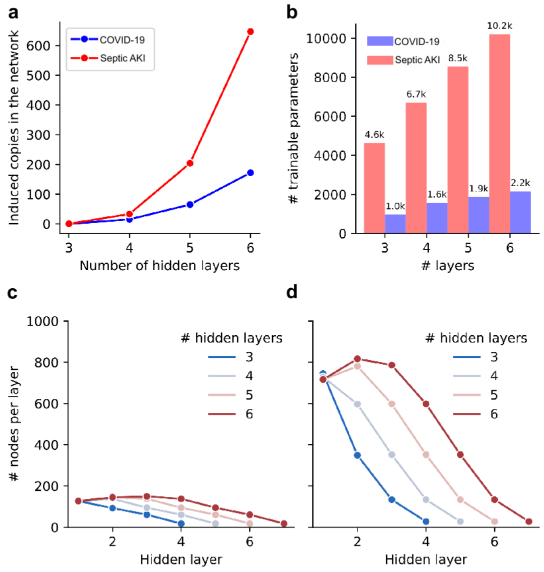

Supplementary Figure 2: Features of the network architecture for the networks generated using the proteomic content of the septic AKI and COVID-datasets. **a)** If the number of layers of the neural network surpasses the number of nodes in a given pathway, a copy is induced of the final node. Naturally, the number of copies increases as the number of hidden layers in the network increases. **b)** The number of trainable parameters in the network is in the thousands, and increases with the number of layers. The COVID-BINN has fewer trainable parameters than the AKI-BINN, due to the lower number of proteins in the dataset. **c)** The number of nodes in the COVID-BINN when created with varying number of layers. The y-axis depicts the number of nods per layer. **d)** The number of nodes in the sepsis-BINN when created with varying number of layers.

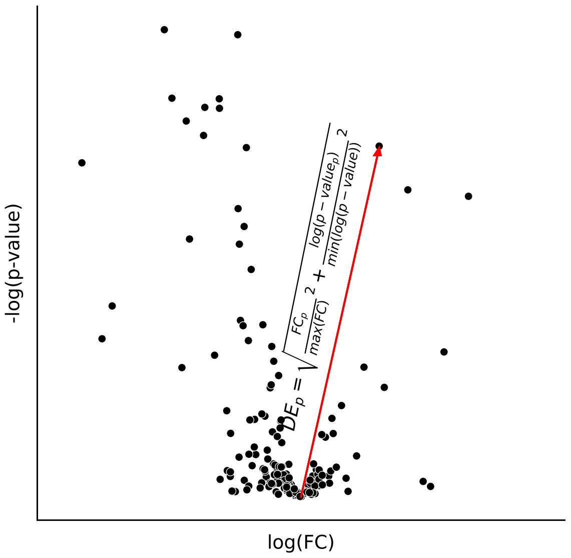

Supplementary Figure 3: Visualization of the DE-score. To get a quantitative measure of level of differential expression, the DE-score was devised. The score can be seen as the Euclidean distance from *origo* to a protein in the volcano plot.

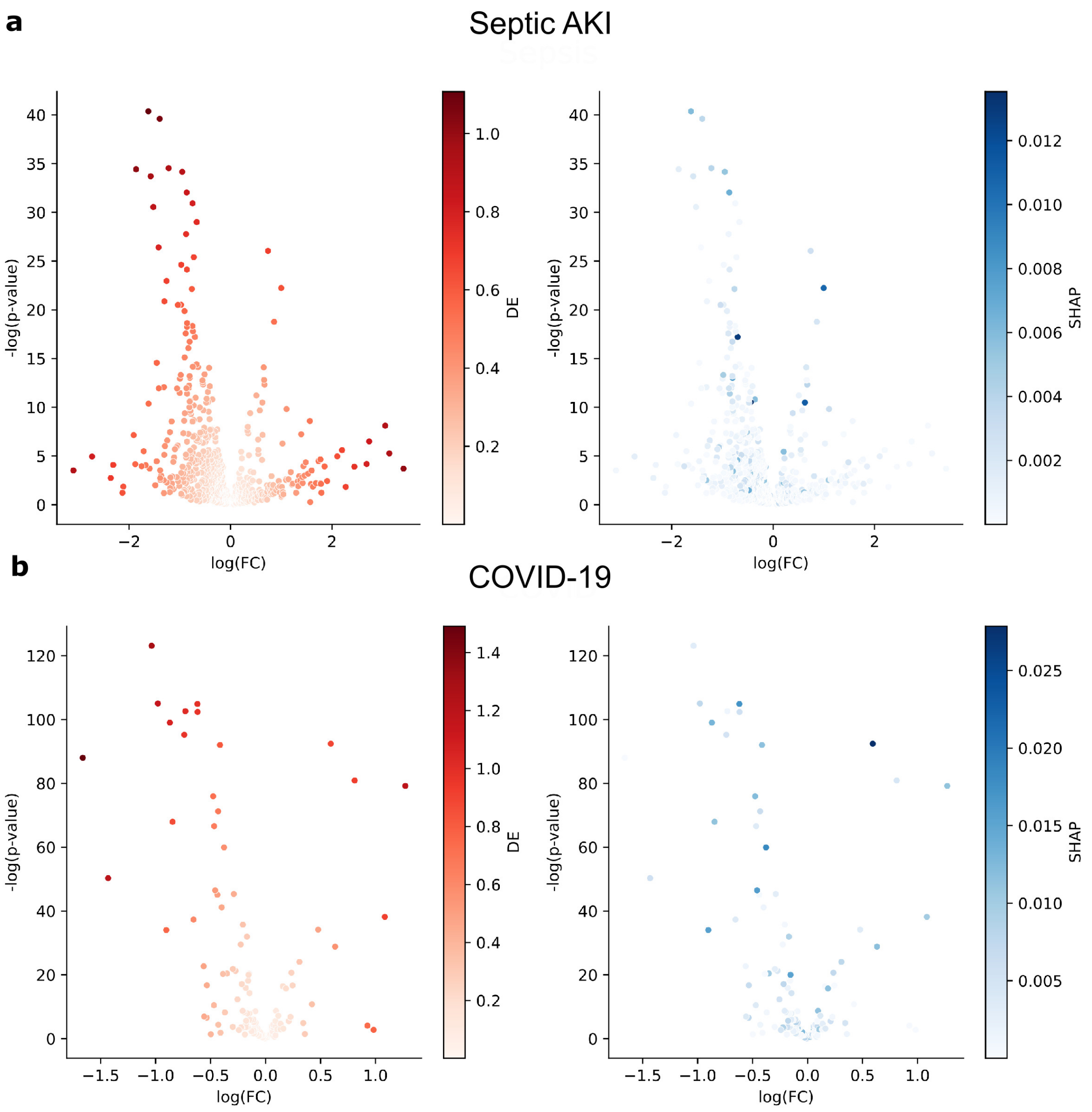

Supplementary Figure 4: Volcano plots of the septic AKI and COVID-dataset colored by DE and adjusted SHAP value. a) The volcano plots for the septic AKI dataset. **b)** The volcano plots for the COVID-dataset. Coloring the volcano plots by DE-score (left) demonstrates how the most important proteins are selected by level of differential expression. The *p-values* are calculated using linear least-squares regression and multiple testing correction is done using the Benjamini-Hochberg-method [1].

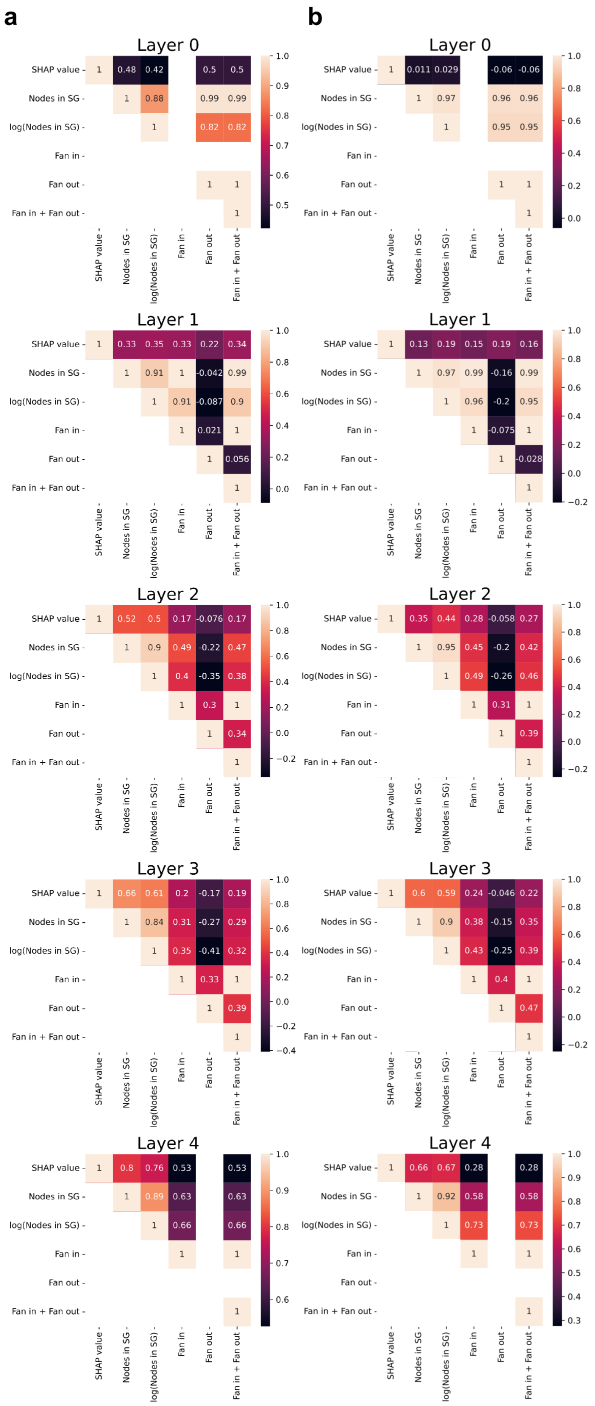

Supplementary Figure 5: Correlation between graph features and SHAP value. Different graph attributes were correlated against each other and against the SHAP value. **a)** The correlation between graph features and SHAP value for the AKI-BINN. **b)** The same but for the COVID-BINN. Nodes in a subgraph $(N_{SG}, log(N_{SG}))$ has the strongest correlation with SHAP value in most layers.

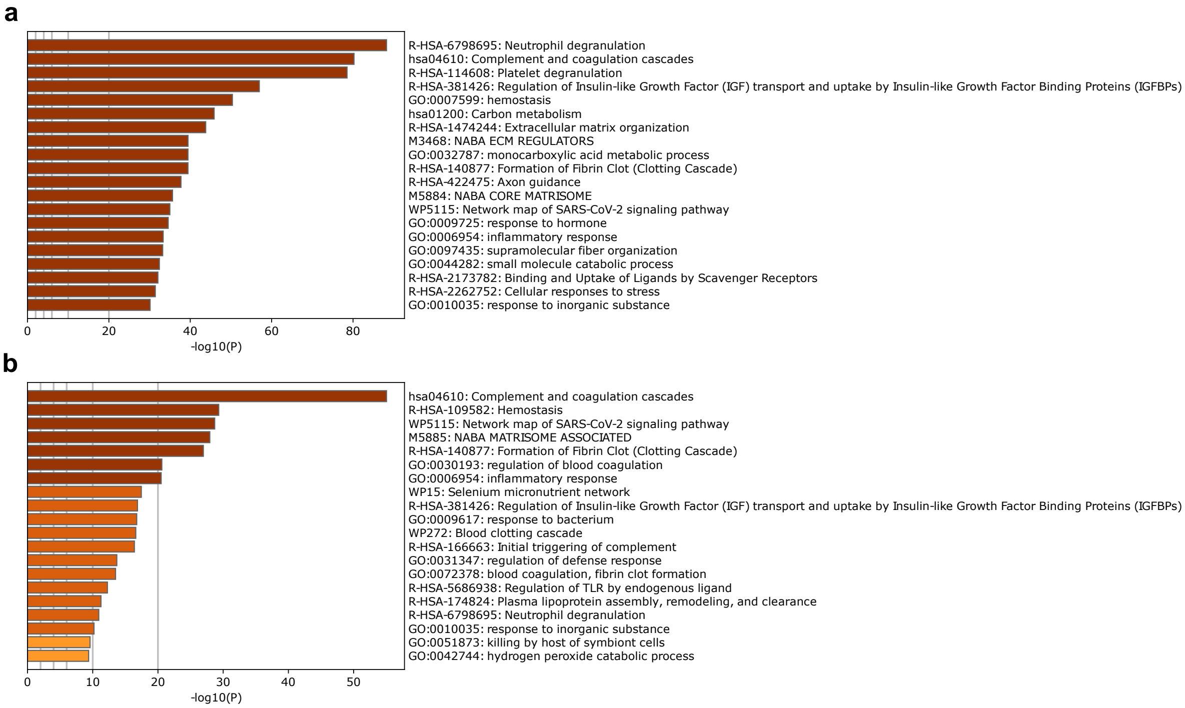

Supplementary Figure 6: Pathway analysis of the septic AKI and COVID-dataset using Metascape. The proteomic content was analyzed by Metascape [2] and the 20 pathways with the lowest *p-value* are shown. Metascape utilizes the hypergeometric test as well as Benjamini-Hochberg correction algorithm to calculate *p-values*. **a)** The top pathways identified in the sepsis-dataset. **b)** The top pathways identified in the COVID-dataset. There is a large overlap in the pathways and processes which are highlighted in the datasets, many of them relating to immunity and inflammation, such as the *complement cascade* and the *inflammatory response*.

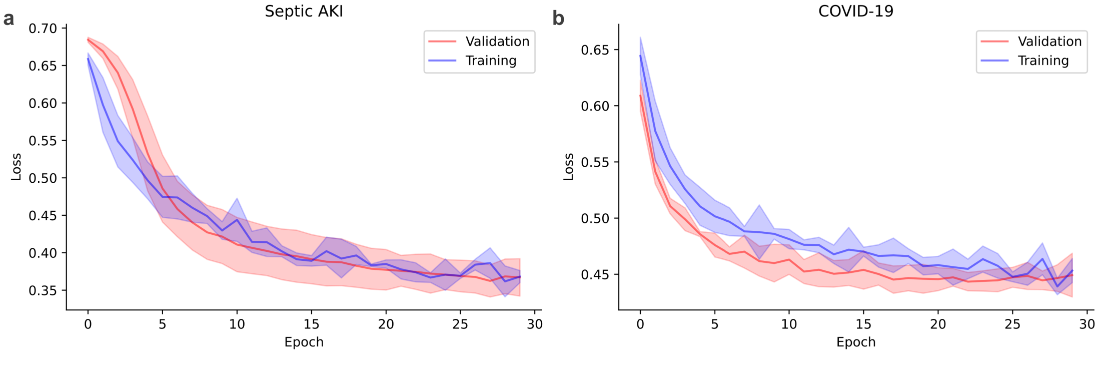

Supplementary Figure 7: Training and validation loss for the BINNs. Training and validation losses were recorded during k-fold cross validation ($k = 3$). **a)** The losses over 30 epochs for the AKI-BINN. **b)** The losses over 30 epochs for the COVID-BINN. If the loss is greater during validation than during training, the model is over-fitting. As can be seen, for both models, over-fitting does not occur. For the COVID-19 data, the model is under-fitting. The loss is calculated using the cross-entropy function. For both **a** and **b** the errorbands show the 95% confidence interval.

Supplementary References

1. Benjamini, Y. & Hochberg, Y. Controlling the False Discovery Rate: A Practical and Powerful Approach to Multiple Testing. *Journal of the Royal Statistical Society: Series B (Methodological)* **57,** 289–300. https://doi.org/10.1111%2Fj.2517-6161.1995.tb02031.x (Jan. 1995).

2. Zhou, Y. *et al.* Metascape provides a biologist-oriented resource for the analysis of systems-level datasets. *Nature Communications 2019 10:1* **10,** 1–10. https://www.nature.com/articles/s41467-019-09234-6 (Apr. 2019).
