# Supplementary material for: Interpreting biologically informed neural networks for enhanced proteomic biomarker discovery and pathway analysis
Source: Nat Commun. 2023 Sep 2;14:5359. doi: 10.1038/s41467-023-41146-4 (PMC10475049; doi:10.1038/s41467-023-41146-4)
Supplement: Supplementary file 3 — Reporting Summary [file 41467_2023_41146_MOESM3_ESM.pdf]

nature portfolio

Corresponding author(s): Johan Malmström, Erik Hartman

Last updated by author(s): Aug 2, 2023

Reporting Summary

Nature Portfolio wishes to improve the reproducibility of the work that we publish. This form provides structure for consistency and transparency in reporting. For further information on Nature Portfolio policies, see our Editorial Policies and the Editorial Policy Checklist.

Statistics

For all statistical analyses, confirm that the following items are present in the figure legend, table legend, main text, or Methods section.

n/a	Confirmed	
[ ]	[x]	The exact sample size (*n*) for each experimental group/condition, given as a discrete number and unit of measurement
[ ]	[x]	A statement on whether measurements were taken from distinct samples or whether the same sample was measured repeatedly
[ ]	[x]	The statistical test(s) used AND whether they are one- or two-sided<br>*Only common tests should be described solely by name; describe more complex techniques in the Methods section.*
[x]	[ ]	A description of all covariates tested
[ ]	[x]	A description of any assumptions or corrections, such as tests of normality and adjustment for multiple comparisons
[ ]	[x]	A full description of the statistical parameters including central tendency (e.g. means) or other basic estimates (e.g. regression coefficient) AND variation (e.g. standard deviation) or associated estimates of uncertainty (e.g. confidence intervals)
[ ]	[x]	For null hypothesis testing, the test statistic (e.g. *F*, *t*, *r*) with confidence intervals, effect sizes, degrees of freedom and *P* value noted<br>*Give P values as exact values whenever suitable.*
[x]	[ ]	For Bayesian analysis, information on the choice of priors and Markov chain Monte Carlo settings
[x]	[ ]	For hierarchical and complex designs, identification of the appropriate level for tests and full reporting of outcomes
[ ]	[x]	Estimates of effect sizes (e.g. Cohen's *d*, Pearson's *r*), indicating how they were calculated

Our web collection on statistics for biologists contains articles on many of the points above.

Software and code

Policy information about availability of computer code

Data collection	MS data was collected using an Q Exactive HF-X.
Data analysis	The source-code developed in this project is made available through GitHub (https://github.com/InfectionMedicineProteomics/BINN). The software used to process the MS-data were: OpenSwath (2.6), GPS (0.0.1) (https://github.com/InfectionMedicineProteomics/gps), DPKS (0.1.1) (https://github.com/InfectionMedicineProteomics/DPKS). The following Python-packages were used in developing the project: keras (2.9.0), matplotlib (3.5.2), numba (0.55.2), numpy (1.21.0), pandas (1.4.3), plotly (5.10.0), scikit-learn (1.1.1), scipy (1.8.1), seaborn (0.11.2), shap (0.41.0), torch (1.12.0).

For manuscripts utilizing custom algorithms or software that are central to the research but not yet described in published literature, software must be made available to editors and reviewers. We strongly encourage code deposition in a community repository (e.g. GitHub). See the Nature Portfolio guidelines for submitting code & software for further information.

Data

Policy information about availability of data

All manuscripts must include a data availability statement. This statement should provide the following information, where applicable:

- Accession codes, unique identifiers, or web links for publicly available datasets
- A description of any restrictions on data availability
- For clinical datasets or third party data, please ensure that the statement adheres to our policy

All the data used in this manuscript was either downloaded from open repositories or has been uploaded to such repositories and are publicly available. The

previously published DIA-MS septic AKI dataset is available with the PRIDE accession code PXD038394. The COVID-19 dataset is available with the PRIDE accession code PXD025752. The Olink dataset is available from: https://doi.org/10.6084/m9.figshare.20260998.v1. The previously unpublished DIA-MS septic AKI dataset has been deposited to the ProteomeXchange Consortium via the PRIDE partner repository with the identifier PXD044264. The Reactome Pathway Database was downloaded from: https://reactome.org/download-data in July 2022.

Human research participants

Policy information about studies involving human research participants and Sex and Gender in Research.

Reporting on sex and gender	Analyses based on sex and/or gender was not included in this study.
Population characteristics	Patient demographics, medical history, severity scores, length of stay, physiologic data and hospital mortality were collected from the Finnish Intensive Care Consortium prospective database (Tieto Ltd, Helsinki, Finland) with a study-specific case report form. From original study (Meri Poukkanen et al. 2013 Hemodynamic variables and progression of acute kidney injury in critically ill patients with severe sepsis: data from the prospective observational FINNAKI study).
Recruitment	AKI status was screened at admission and during the first 5 days of ICU stay.
Ethics oversight	The AKI samples were processed in accordance with the Helsinki Declaration. The Ethics Committee of the Department of Surgery, Helsinki and Uusimaa Hospital District, approved the study protocol and each participant or their proxy gave written informed consent. The Ethics Committee of the Department of Surgery, Helsinki and Uusimaa Hospital District, also approved the inclusion of participants for all centers involved as well as the use of deferred consent (Reference Number 18/13/03/02/2010). Patient demographics, medical history, severity scores, length of stay, physiologic data and hospital mortality were collected from the Finnish Intensive Care Consortium prospective database (Tieto Ltd, Helsinki, Finland) with a study-specific case report form.

Note that full information on the approval of the study protocol must also be provided in the manuscript.

Field-specific reporting

Please select the one below that is the best fit for your research. If you are not sure, read the appropriate sections before making your selection.

☒ Life sciences ☐ Behavioural & social sciences ☐ Ecological, evolutionary & environmental sciences

For a reference copy of the document with all sections, see nature.com/documents/nr-reporting-summary-flat.pdf

Life sciences study design

All studies must disclose on these points even when the disclosure is negative.

Sample size

Sample sizes of previously published data were determined in previous studies as the datasets were downloaded from existing repositories.

For the septic AKI data, Scott et al. reports the following: "Overall, 141 samples were chosen for up to 5 time points from 23 acute kidney injury patients. The patients were from two distinct subphenotypes that were previously defined using a panel of clinical markers and latent class analysis. No power analysis was performed, the 23 patients were selected on the basis of culture-positive sepsis."

Aaron M. Scott et al. Generalized pre-
cursor prediction boosts identification rates and accuracy in mass spectrometry
based proteomics. Communications Biology, 6(1), June 2023

For the COVID-19 samples from the Charité hosptial: all samples reported in Demichev et al. were retrieved from the observational study Pa-COVID-19. No samples were excluded and no power calculation was performed to determine dataset size.

For the COVID-19 samples from the Innsbruck hosptial: all 99 samples were gathered in Demichev et al. and included based on criteria included in Demichev et al.

Demichev V. et al. A time-resolved proteomic and prognostic map of COVID-19.
Cell Systems, 12(8):780–794.e7, 2021

Kurth, F. et al. (2020). Studying the pathophysiology of coronavirus disease 2019: a protocol for the Berlin prospective COVID-19 patient cohort (Pa-COVID-19). Infection, 48(4), 619–626

For the olink-data: Batra et al. report no statistical methods used to predetermine sample size. Dataset size was therefore based on availability.

Richa Batra et al. Urine-based multi-omic comparative analysis of COVID-19
and bacterial sepsis-induced ARDS. Molecular Medicine, 29(1), January 2023

	Sample sizes for the newly published septic AKI dataset were decided based on the availability of the data used in the study, (ie. culture positive sepsis) and based on available sample labels (whether or not samples had been stratified to subphenotypes).
Data exclusions	No data was excluded.
Replication	The software was validated on three independent datasets with two testing datasets and was succesful indepdent of dataset.
Randomization	Septic AKI-data: two datasets are included here and used for training and testing respectively. For both datasets, samples are allocated into two severity categories based on latent class analysis (previously published). The training dataset was previously published (Nisula et al.), and used for training/validation of the algorithm. Thereafter new samples were analyzed and used as an independent testing cohort. During trianing-validation, k-fold cross-validation is performed, where the data is divided into splits randomly. In such cases a stratified split was chosen so that each split had roughly the same proportion of less/more-severe samples.<br><br>Nisula, S. et al. Incidence, risk factors and 90-day mortality of patients with acute kidney injury in Finnish intensive care units: the FINNAKI study. Intensive Care Medicine 39, 420–428. https://doi.org/10.1007/s00134-012-2796-5 (Jan. 2013).<br><br>COVID-data: There are two COVID-datasets, published in the same study but gathered at different hospitals (Demichev et al.). All patients were categorized into one of seven severity categories according to the WHO standard. Severity 6 and 7 can be considered highly severe, as they require mechanical ventilation. We defined these two categories as "more severe" and severity 1-5 as "less severe". Samples gathered at the Charité hosptial was used for training/validation (same procedure as above) and samples gathered at the Innsbruck hosptial for testing.<br><br>Demichev, V. et al. A time-resolved proteomic and prognostic map of COVID-19. Cell Systems 12, 780–794.e7. issn: 2405-4712 (2021).<br><br>Olink-data: No testing set was included, and all the previously published samples were used for training and validation (same procedure as above). Dataset is described in original study (Batra et al.).<br><br>Batra, R. et al. Urine-based multi-omic comparative analysis of COVID-19 and bacterial sepsis-induced ARDS. Molecular Medicine 29. https://doi.org/10.1186/s10020-023-00609-6 (Jan. 2023)
Blinding	Blinding was not applied to this study as we use machine learning algorithms which require knowledge of ground truth to be able to train.

Reporting for specific materials, systems and methods

We require information from authors about some types of materials, experimental systems and methods used in many studies. Here, indicate whether each material, system or method listed is relevant to your study. If you are not sure if a list item applies to your research, read the appropriate section before selecting a response.

Materials & experimental systems

n/a	Involved in the study	
☒	☐	Antibodies
☒	☐	Eukaryotic cell lines
☒	☐	Palaeontology and archaeology
☒	☐	Animals and other organisms
☒	☐	Clinical data
☒	☐	Dual use research of concern

Methods

n/a	Involved in the study	
☒	☐	ChIP-seq
☒	☐	Flow cytometry
☒	☐	MRI-based neuroimaging
